# Exploring the acceptability, feasibility, and appropriateness of a communication-friendly classroom tool for use in Irish schools: A qualitative inquiry

**DOI:** 10.1371/journal.pone.0287471

**Published:** 2023-06-22

**Authors:** Aoife Lily Gallagher, Rachel Murphy, Johanna Fitzgerald, Carol-Anne Murphy, James Law

**Affiliations:** 1 Faculty of Education and Health Science, Health Research Institute, University of Limerick, Limerick, Ireland; 2 Faculty of Education and Health Science, University of Limerick, Limerick, Ireland; 3 Faculty of Education, Mary Immaculate College, Limerick, Ireland; 4 School of Education, Communication and Language Sciences, Newcastle University, Newcastle upon Tyne, United Kingdom; The University of Alabama, UNITED STATES

## Abstract

**Background:**

Ten percent of the school-aged population have speech, language, and communication needs (SLCN) that impact access to the curriculum. Successful implementation of classroom-based SLCN interventions can reduce barriers to learning, thereby improving educational outcomes for this vulnerable population. The challenges of implementing innovations in educational settings are well-documented, yet limited studies have addressed such considerations when developing, and piloting universal level SLCN interventions for use in Irish schools.

**Methods:**

A qualitative exploratory study was undertaken to establish the acceptability, feasibility, and appropriateness of a universal level SLCN intervention. An advisory panel of teachers (n = 8) and children with SLCN (n = 2) were engaged as co-researchers in the study. The Communication Supporting Classrooms Observation Tool, developed as part of the Better Communication Project in the UK, was trialled across a diverse sample of school settings (n = 5). Semi-structured interviews were conducted with school practitioners and school leaders, and a deductive content analysis was undertaken using the domains of the Consolidation Framework for Implementation Research.

**Discussion:**

The observation tool was viewed as acceptable with suggested additions. Integrating use of the tool within existing data-informed, school self-evaluation processes aimed at supporting school improvement was noted as a potential means of supporting implementation. A knowledge gap in relation to school-based models of support for SLCN was identified which may negatively impact implementation. An implementation strategy targeting coherence, cognitive engagement and contextual integration is indicated if the tool is to be normalised into routine practice in Irish classrooms. Implementation needs appeared to vary at the school level.

**Conclusions:**

The importance of early-stage exploration to guide implementation planning with regards to developing and testing universal level interventions for SLCN in schools is highlighted. Engaging an advisory panel provides important insights to guide implementation decisions. Findings suggest an adaptive design is required when planning implementation studies targeting classroom setting.

## Introduction

According to findings from international studies, approximately 10% of children in every classroom present with [1,2]. Whilst limited robust prevalence and service data exists about children and young people with speech, language, and communication needs (SLCN) of school-age in Ireland, findings from studies that have been conducted in the Irish context suggest that children with SLCN are disproportionately under-identified during the school years [3], and that there may be limited knowledge and understanding of these needs amongst school practitioners [4].

Childhood SLCN can occur in association with a bio-medical condition, because of impoverished language exposure, or with no obvious cause as a part of a neurodevelopmental disorder [5]. Difficulties may occur in comprehension and expressive language and can impact negatively on the development of literacy and socialization [5,6] As teaching, learning and assessment is primarily language-based, individuals with SLCN can experience many barriers to accessing the curriculum [7,8], resulting in poor educational attainment [9–11]. Lifelong negative sequelae of unmet SLCN are well-documented, and include increased risk of social isolation, poor mental health, and reduced employability [12,13].

Prior to school entry, interventions targeting SLCN are delivered by a speech and language therapist (SLT) typically in primary healthcare settings. At school-age, SLCN becomes the responsibility of both the SLT and teacher, who must work collaboratively to plan and deliver SLCN interventions in school [14,15]. To address the needs of individuals with SLCN in Ireland, three distinct tiers of intervention exist; interventions delivered at a *universal level* (support for all); interventions delivered at a *targeted level* (support for some); and interventions delivered at a *specialist level* (support for few) [16,17]. This tiered approach, underpinned by public health and response-to-intervention educational principles [18–20], ensures early and accurate identification of needs, more equitable access to appropriate support, and cost-effective allocation of specialist resources [21,22].

Successful implementation of SLCN interventions at the universal level is an essential component of the tiered model of delivery [21,23]. Integration of SLCN methods into routine teaching practice can reduce language barriers to learning and assessment. The response of individual students to class-based interventions can be monitored and can inform resource allocation decisions for more expensive targeted and specialist level interventions.

An SLT working in school will deliver interventions across all levels of support. At the specialist level, they will deliver interventions directly to the child, whereas at targeted and universal levels, much of the work of the SLT is akin to the role of implementation facilitator; that is, working closely with school practitioners to deliver SLCN interventions through education and coaching [23,24].

To date, research for school-aged children with SLCN has focused more on specialist level SLCN interventions, rather than interventions at a universal level [24,25]. Issues of feasibility, acceptability and relevance of SLCN interventions in relation to their use in the school setting are reported in the literature [26,27], with evidence to suggest that such issues have resulted in reduced treatment fidelity, and in turn, less favorable outcomes for children with SLCN [28–30].

### Implementation science in communication sciences research

A vast range of evidence-based programmes have been developed for use in schools for children with special educational needs, and successful implementation of such interventions is acknowledged to be fraught [31,32]. Implementation science is a field of inquiry which allows the study of methods to support the systematic uptake of evidence into routine practice [33]. The potential of implementation science as a means of supporting the uptake of evidence-based programmes in schools is well-established [33–35]. Most school-based implementation studies have targeted emotional behavioral difficulties [34,35], literacy needs [36], and/or autism [37,38].

Whilst the potential of implementation science in the field of communication science has been discussed for some time [39,40], the application of implementation frameworks and theories in research is in its infancy. In a recent scoping review, researchers reported that the majority of studies addressing implementation considerations focused on interventions for adult populations in hospital settings, with only eight percent of studies focused on SLCN interventions in educational settings [41]. Of those, two studies involved early years settings, and the remaining three studies did not report the use of an explicit implementation science framework. A scoping review is currently being undertaken to map the use of implementation science concepts and frameworks that may guide the development of universal level school-based SLCN intervention research [42].

Successful implementation of SLCN interventions at a universal level presents unique challenges. The desired outcome when addressing such a foundational skill as language and communication is not necessarily the delivery of a discrete programme with sufficient fidelity for a requisite number of hours, across a defined number of weeks, but is often sustained changes in teacher interactional patterns and language use throughout the school day and across teaching, learning and assessment tasks. Such changes go to the very heart of the craft of teaching. In terms of the intervention itself, there can be a cross-sectoral ‘task shift’ element at play, whereby techniques and methods developed in one field (health) are to be implemented by actors from another (education), potentially exacerbating issues of fit [43,44]. There are also several well-documented barriers related to the role of the SLT as implementation facilitator in schools. These barriers relate to professional differences in perspectives about SLCN [45–47], in addition to many logistical and systems level challenges [43,44].

### The ‘communication supporting classroom observation tool’

The ‘Communication Supporting Classrooms Observation Tool’ (CSCOT) was developed in the UK as part of a national government-funded research project, the Better Communication Research Project, and was co-led by JL [48,49]. See S1 File for a copy of the tool. The CSCOT is an observational booklet that practitioners can use to reflect on three core aspects of the classroom setting related to speech, language, and communication. These include: (a) the language learning environment (i.e., the physical environment and learning context); (b) language learning opportunities (i.e., the structured opportunities that are present in the setting to support language development) and (c) language learning interactions (i.e., the ways in which adults speak to the children within the setting).

Each section includes a list of strategies that are shown to be effective in supporting communication. The tool allows the observer to record their observations in relation to the three language learning dimensions. For example, in relation to the language learning environment, it can be noted whether certain elements that promote language and communication are present. In terms of language interactions, the number of times (out of five) that these behaviours were observed can be recorded. There is also an option to note any additional comments in relation to the dimensions.

The tool is underpinned by social constructivist learning theory which holds that children learn language best by using naturalistic methods in their everyday environment whereby the adult scaffolds communicative opportunities within their zone of proximal development [50]. In the context of this study, the teacher is the actor who will implement the tool and techniques such that the child has increased opportunities to produce verbal output, and to gain effective feedback to support their language development.

Our reasons for choosing to trial this tool were threefold: first, the tool was developed based on high quality research evidence [51–53]; second, the tool has been previously trialed in UK primary schools in early years classrooms up to year 2, with positive outcomes [54]; and third, the focus of the intervention in adjusting interaction in the classroom was a priority identified in our previous work with children and young people with SLCN [46].

As many elements within the core dimensions of the tool are not age-specific but more general strategies to support communication, we took the view that it was worth exploring the acceptabilty, feasibility and appropriateness of the tool for use beyond the original early years classroom settings that it had been trialed in. We therefore recruited practitioners from across any primary school years which include children aged 5 years to 12 years. Given the lack of tools available for use in post-primary classrooms to support communication [24,48], we decided to further extend the study to include these settings also. Post primary schools include children aged 12 to 18 years.

### Research questions

Our research questions were as follows:

1. How acceptable, feasible, and appropriate is the CSCOT from the perspective of speech and language therapists, teachers and school leaders working in Irish schools?
2. What changes, if any, are required to the tool to enhance the acceptability, feasibility, and appropriateness of the tool for use in school settings in Ireland?
3. What contextual considerations are important in planning future research in relation implementation of the CSCOT in the Irish school context?

We defined *acceptability* as the perceptions of implementation stakeholders (i.e., teachers, SLTs, and school leaders) regarding whether the tool is agreeable or satisfactory. We defined *appropriateness as* the perceived fit, relevance, and/or the compatibility of the tool for use in Irish schools. By *feasibility*, we meant the extent to which the tool could be successfully used or carried out within the school settings [55]. Implementation strategies were taken to be active, targeted methods and techniques needed to facilitate adoption, implementation, and sustainment of the tool such that it can become ‘normalized’ or embedded into routine practice [56].

## Methods

### Study design

A qualitative description study was undertaken [57], using directed content analysis [58]. The qualitative study was a ‘current situation’ exploration [59], with the aim of using the findings to inform the planning of a future pilot study. The research team included two female white lecturers in speech and language therapy (ALG, CAM), one female lecturer in education (JF), one white female research assistant (RM), and one white male lecturer in speech and language therapy (JL). Analytical records were kept by members of the team in order to reflect on the ways in which their beliefs, and values influenced their interpretation of the data.

The study included public patient involvement. Teachers and children with SLCN were invited to be part of the study in the role of co-researchers to provide guidance to the research team about the suitability of the tool prior to the trial, and at the end of the study to provide views based on the findings as to whether the tool should be further tested [60].

Two advisory panels were set up. One panel comprised children and young people with SLCN of primary age, and of post-primary age (*n* = 2). The second panel included teachers from primary and post-primary settings (*n* = 8). Given the importance of addressing power imbalances in facilitating authentic engagement with the children and young people with SLCN [61,62], the two groups operated independently. Meetings were held online using a humble inquiry approach [63] and were facilitated by ALG and RM.

No substantive changes were recommended by the advisory panels prior to trialing the tool. The children with SLCN were particularly in favour of the elements of the tool that involved peer to peer opportunities to use language in classroom. Post-primary teachers were of the view that most elements of the language learning environment section would not be considered relevant for use in their setting, and that some minor changes to the language of the other sections may be required.

### Methodological assumptions

The underpinning assumptions of this study are aligned with subjectivist epistemology, that is; a recognition that the subjectivity of the researcher is intimately involved in the research process. Within this paradigm, however, we take the position that objectivity is an ideal that we can strive to attain by enhancing transparency and credibility of the research. For example, we used a database to track coding decisions and kept analytical memos. We held analysis meetings as a research team to interrogate the data, exploring alternative lines of reasoning, and to identify areas for critical reflection [61,64]. We reported the study in accordance with the Standards for Reporting Qualitative Research criteria [65].

### Ethics

Ethical approval was granted a priori for this study by the Faculty of Education and Health Sciences’ Human Research Ethics Committee, at the University of Limerick (ref: 2020_05_10). Written consent was obtained from the participants in the study.

### Setting, sampling, and recruitment

The Irish education system is made up of primary school and post-primary school (also known as secondary school). There are approximately 3000 schools in the Republic of Ireland. Typically, children start primary school at age 5 years and post-primary level at 12 years. In primary school, students are taught by one teacher who teaches across all subjects, whereas in post-primary settings, students are taught by different subject teachers.

To gain a comprehensive understanding of the interplay of factors that might influence implementation of CSCOT in Irish schools, and to be able to match barriers to specific implementation strategies [66], a maximum variation purposeful sampling strategy was employed [67]. We recruited schools by education level (primary and post-primary level), location (urban and rural settings), and socio-demographic needs which were identified by Delivering Equality of Opportunity in Schools (DEIS) status. DEIS forms part of the Department of Education social inclusion strategy to help children and young people who are at risk of or who are experiencing educational disadvantage in the Irish educational system. Information leaflets and posters were circulated about the study via established teaching networks (*n* = 4), professional teaching bodies (*n* = 2) and via social media (twitter). Within each school, we sought to recruit a wide range of professionals who work within the Irish education system. In schools where a speech and language therapist visited regularly, we aimed to recruit them to trial the use of the CSCOT with a teacher.

### Materials

As described, the CSCOT is used by a pair of practitioners to identify changes in the classroom environment to optimise communication and includes guidance on what to observe, with examples and explanations of each item. An electronic copy was provided to participants, with the option of printing a paper copy. Practitioners decided when and with whom they would trial the tool with. Participants were sent a short instructional video on using the tool and were invited for a virtual discussion with the lead investigator (ALG) to address any queries prior to the study. Participants trialed use of the tool during a 40-minute lesson, and then met to discuss their findings to identify changes in their practice that might optimise communication for the children in their classroom.

### Data collection

A short online questionnaire was developed using the Qualtrics survey tool to gather background data about participants. The questionnaire, completed in February 2021, included 10 questions related to each participant’s current role, school setting (size, number of students), years of professional experience, and their level of qualifications. The CSCOT was trialed by participants between March 2021 and June 2021. Post trial, participant dyads attended an online interview. The interviews lasted approximately 40 minutes and were facilitated online via Microsoft Teams. ALG facilitated the practitioner interviews, and JF facilitated interviews with school leaders. RM acted as observer. Topic guides were developed in advance and shared with the participants (see S2 File for questionnaire and topic guides).

### Data analysis

To guide data collection and analysis we used the domains of the Consolidated Framework for Implementation Research (CFIR) [68]. The CFIR is a meta-theoretical framework developed to create a consistent vocabulary for domains and constructs in implementation science research and has been utilized extensively in implementation science studies in a variety of research contexts. The CFIR includes thirty nine constructs organised into five domains: outer setting, inner setting, characteristics of the individual, intervention characteristics, and process. Outer setting includes external influences on an organization such as policies and incentives, whereas inner setting includes influential factors related to the structure, culture, and resources of an organization. The domain characteristics of the individual includes factors related to the implementor that may influence implementation such as self-efficacy, and knowledge and beliefs about an innovation. Characteristics of the intervention encompasses factors related to the innovation itself such as adaptability, trialability, complexity, and cost. Process includes factors that would support successful implementation of an innovation for example whether or not there is a formally appointed internal leader involved in implementation of an innovation.

ALG, RM and JF were involved in transcription and analysis. Automatic transcripts of the interviews were generated via Microsoft Teams, and checked for accuracy by RM and ALG. Aligned with the underpinning the assumptions of this subjectivist study and our position within this paradigm that objectivity is an ideal that we can strive to attain by enhancing transparency and credibility, coding was undertaken independently by two researchers (ALG and RM), and all analytical decisions between researchers were tracked via the recording of analytical notes on NVivo (V 12.1).

Open codes were then organised using the domains of the CFIR. Three analysis meetings were held to interrogate the findings of the analysis, and to agree the final codes (ALG, JF and CAM). Points of discussion in analytical decisions within the team centred around differences in terminology across education and health, and were resolved through dialogue, and by cross-checking definitions of the CFIR in relation to the codes.

A summary of the findings in relation to the intervention characteristics was presented to the advisory panels by ALG and RM once the analysis was complete.

## Results

### Sample characteristics

The tool was trialed in five school sites across the Republic of Ireland: three primary and two post-primary schools. One school was classified as DEIS (Delivering Equality of Opportunity in Schools), a programme introduced in Ireland in 2006 aimed at providing additional resources to schools with high proportions of students from socioeconomically disadvantaged backgrounds who are at risk of educational failure. Characteristics of the schools included in the study are presented in Table 1.

**Table 1:** School characteristics.

School Reference	Type	Setting	Sex	Location	N Pupils	SLT^a^ service	DEIS^b^
S1	Primary	Urban	Mixed	Leinster	285	No	No
S2	Primary	Urban	Mixed	Leinster	400	Yes	No
S3	Post-primary	Urban	Mixed	Munster	1100	No	No
S4	Primary	Urban	Girls	Munster	220	No	No
S5	Post-primary	Urban	Girls	Leinster	165	No	Yes
S6	Post-primary	Rural	Boys	Munster	230	No	No

^a^ SLT = speech and language therapy.

^b^ DEIS is a school designated as serving a population of high socio-economic needs.

Participants included: school leaders (*n* = 3), class teachers (*n* = 5), SLTs (*n* = 1), special education teachers (*n* = 2), and special needs assistants (*n* = 1). Seven participants had undertaken formal studies at a postgraduate level in addition to their professional teaching qualification. Professional experience in the sample ranged from 5 years to 22 years. Further participant details are outlined in Table 2.

**Table 2:** Participant characteristics.

School code	Participant code	Role	Sex	EQF^a^ Level of qualification
S1	P1	Deputy Principal	Male	Level 7
S1	P2	Teacher	Female	Level 7
S2	P3	SLT	Female	Level 7
S2	P4	Special Class Teacher^b^	Female	Level 7
S3	P5	SENCO^c^ & Deputy Principal	Female	Level 7
S3	P6	Teacher	Female	Level 7
S4	P7	Teacher	Female	Level 7
S4	P8	Teacher	Female	Level 6
S5	P9	SNA^d^	Female	Level 6
S5	P10	Teacher	Female	Level 6
S5	P11	Principal	Male	Level 6
S6	P12	Deputy Principal	Female	Level 6

^a^ EQF = European Qualification Framework.

^b^ A special class teacher is a teacher that is responsible for teaching in a class attended by students with more complex special educational needs. The class typically involves a small class size and is located within a mainstream school setting.

^c^ SENCO = special educational needs coordinator is a teacher usually tasked with leadership and management of special education provision in schools.

^d^ SNA = special needs assistants are employed to address the medical and/or care needs of children and young people in school.

### ‘Communication supporting classroom observation tool’

Five key concepts were identified across the dataset related to characteristics of the tool: (a) advantages; (b) relevance; (c) complexity; (d) adaptability; and (e) affective and relational factors. See Table 3 for illustrative quotes in relation to intervention characteristics.

**Table 3:** Participant views of the CSCOT for Use in Irish schools.

Intervention characteristics	Descriptive summary of findings	Illustrative quotes
**Advantages**	Opportunities for conversations about language and communication	“There is definitely a lot of classrooms with different children where it would be a great starting point to open up a conversation or to have that kind of like starting point for planning or goal setting around communication. . .” (P10, subject teacher, post-primary)
	Effective tool for reflecting on practice	“Yeah, I see it as more of a teacher self-reflection tool. So, it’s more of a reflection of your own practice…Like it would help in my own planning in how I write my lesson plans. Becoming aware of my own practice and how I can improve myself as a teacher. That would be where I’d see myself using it” (P8, class teacher, primary)
	Potential to support collaborative relationships	“I think it was a very positive experience. And really helpful to be kind of highlighting the importance of supporting language across settings. And to kind of have that shared language between us and something that was collaborative” (P7, speech and language therapist, special class)
**Relevance**	Items within the language learning interaction section were most relevant, with some recommended additions	“Maybe a bit more on actually explicitly teaching grammar in sentences and things like that… and obviously a class teacher will miss it… when you listen to it every day you can miss it. I don’t think we do enough of the sentence structure work at the moment and teachers don’t know where to start with that” (P7, class teacher, primary)
	The relevance of items related to the use physical spaces is dependent on the individual school setting	“In our school, we (teachers) have our own base classrooms which is brilliant but even with that you are taking in a different cohort each lesson you know? …so, the spaces that are required are very different for every lesson” (P12, deputy principal, post-primary)
**Complexity**	Easy to use, efficient in yielding actionable knowledge for the time spent	“I think in terms of ease of administration, it was pretty easy to go through, the time commitment wasn’t too much” (P2, class teacher, primary)
	Easy to explain which would help when engaging staff in implementing.	“…I’d say I would probably have buy-in from 70% to 80% of staff about this tool if we were to roll it out. Yeah, cos I’d say they would engage…I would only need 20 minutes out of staff meeting to show them” (P10, subject teacher, post-primary)
**Adaptability**	Tool allows for flexible use across learning contexts as determined by the practitioner.	“I’d like ideas or tools like this that I know that I can use in a moment because I think sometimes if you’re too rigid on the plans that you have made then the tail is kind of wagging the dog if you know what I mean, I wouldn’t probably use something if it interfered with me responding to a student or a learning situation” (P7, class teacher, primary)
**Affective/ Relational factors**	The nature of the tool as an observational tool is potentially exposing/ anxiety provoking for teachers, and should be used between practitioners in trusting professional relationships	“So yeah, like I think there’s a massive need for something like this, but I think it would be important that it’s done in a way that is supportive between two people who trust each other. You know on a kind of, you know, level playing field that you know we’re both there to work on communication where it’s not one better than the other” (P5, Special Educational Needs Co-ordinator & deputy principal, post-primary)

#### Advantages

Participants discussed several advantages of the tool. The tool could facilitate opportunities for novel, goal- oriented conversations about language and communication in the classroom. By providing a shared point of reference, it had the potential to strengthen collaborative relationships between teachers and SLTs. Practitioners also noted that the tool could enhance practitioner self-efficacy as it helped to identify and reinforce good practice. Collaborative reflection was viewed as an effective means of improving teaching practice.

#### Relevance

Practitioners across both primary and post-primary school settings identified the language learning interaction items as most relevant. Suggested additions included: (a) the use of questions to optimise communication, (b) supporting peer-mediated interaction, (c) optimising use of language when giving feedback, (d) comprehension checking strategies, (e) reciprocal teaching, and (f) strategies to support more complex grammar use in the classroom. One special education teacher described the need for items that focus on facilitating the use of complex grammar in the classroom.

Views differed about the relevance of items related to the use of space for learning. Post-primary teachers noted the wording of many items in this section as relevant only for early primary school years. Two primary teachers reported that they had little control over the way physical spaces were allocated in their school. Two further participants viewed the use of space as an important influencing factor when optimising communication, suggesting the use of space should go beyond the classroom. For example, one teacher described that they would seek spaces outside of the classroom to enable students participate more actively in drama lessons. Teachers working in post-primary school settings reported that unless they had a base classroom, it was difficult to personalise the space for certain cohorts and/or individual children.

#### Complexity

Participants believed that the tool was easy to use, with clear, culturally-appropriate language. The simplicity of the tool was described as an advantage in order to gain buy-in from peers. Participants indicated that a relatively short amount of time observing a lesson using the tool provided a surprising amount of data to inform lesson planning. One post-primary subject teacher noted that the layout of the tool made it look more time intensive than it was.

#### Adaptability

Teachers across primary and post-primary settings discussed the adaptability of the tool as a positive feature. Adaptability was discussed as a critical characteristic of any innovation in a school setting. Teachers described how they juggled their plans throughout the day and highlighted the need for tools that can be used across whole classroom groups, small peer-led groups, a 1:1 meeting with a student, and both inside and outside the classroom. One teacher stated that if they perceived that an intervention is too prescriptive, they would be highly unlikely to use it.

#### Affective and relational matters

The nature of the relationship between the observer and observee was discussed by participants as an important consideration when using the use of the tool. Participants believed that where there was a relationship of trust and equality, the tool had many advantages. Conversely, they were of the opinion that where a trusting relationship had not yet been established, or where power imbalances exist, the observational element of the tool could feel exposing or unsafe. A special needs assistant discussed initial feelings of anxiety in relation to the observational nature of the tool. Some teachers shared the view that use of the column to record the frequency of teacher communicative behaviours could feel ‘testing.’

### Practitioner level considerations

Three key concepts were identified across the dataset related to practitioner level considerations. These included: (a) self- efficacy, (b) values and beliefs, and (c) knowledge and understanding of SLCN and SEN. See Table 4 for illustrative quotes in relation to practitioner level considerations.

**Table 4:** Illustrative quotes related to practitioner level factors.

Practitioner level factors	Descriptive summary of findings	Illustrative quotes
**Self-efficacy**	A positive approach to change, a belief in one’s own abilities to affect change and a commitment to lifelong learning	“So, if there is a teacher who mightn’t feel very confident about their ability or classroom management it might be a little more difficult” (P4, special education teacher, special class, primary)
	A lack of teaching experience may act as a barrier to implementing the tool	“We have a lot of new teachers. Young teachers. A lot of people mightn’t be brave enough to pick this up. A lot of people wouldn’t say yeah come on in, observe me. They wouldn’t be as okay with it” (P8, class teacher, primary)
**Values and Beliefs**	Practitioners who value collaboration and who routinely work with others in the classroom would be more likely to implement the tool.	“Yes, it would work well in our school. . . For stations because you always have other adults in the room for stations anyway, and then it wouldn’t be intimidating like, then you’re not the only one being watched” (P2, class teacher, primary)
**Knowledge & understanding of SLCN**^**a**^ **& SEN**^**b**^	Practitioners who are not familiar with the continuum of support for children with SEN may not use the tool	“I mean, I have seen withdrawal (from the classroom) for absolutely everything you can think of. . .instead of sorting the classroom…so in that classroom, that is where it won’t be used” (P11, principal, post-primary)
	An awareness and knowledge of SLCN is critical to see the benefit of the tool and to use it.	“Subject teachers probably need more awareness raising around speech and language communication needs in the classroom because we know it’s a hidden disability So, you would have to raise awareness around it for say the maths teacher or the science teacher before they would see the benefit of it. . .” (P10, subject teacher, post-primary)
	Experience of co-practice with an SLT would facilitate implementation	“I think it would nearly have more of a place or more of a need in a setting where an SLT or teacher might work together and be tuned in to all of those strategies. Yeah I think the need for it is huge across settings. But yeah maybe an awareness piece is required first in normal schools” (P8, class teacher, primary)

^a^ SLCN = speech, language, and communication needs.

^b^ SEN = special educational needs.

### Self-efficacy

The most frequently coded construct within this domain was *self-efficacy*. Practitioners reported that given the reflective and potentially exposing nature of the tool, practitioners would need to be confident in their own abilities to use it. One class teacher proposed that if a practitioner had less teaching experience, this may impact their willingness to use the tool. A positive attitude towards change was viewed as necessary by practitioners if the tool was to be implemented on an ongoing basis. Participants reported that i a practitioner was not motivated to improve their own practice, then engagement with reflective processes was unlikely.

#### Values and beliefs

Practitioner values and beliefs were the second most coded construct. Teachers who value professional development and who engage with additional formal and informal learning opportunities would be more inclined to use the tool, as would the teacher who is committed to inclusive pedagogy. Practitioners who value working collaboratively with others, and who engage in the co-delivery of lessons as part of their normal routine would use the tool more readily than those who prefer to work in isolation.

#### Knowledge and understanding of supporting SLCN in school

Knowledge of special educational needs policy, an understanding of the continuum of support model, and an understanding of the nature and impact of SLCN on learning were identified as essential by participants if the tool were to be used in school. Participants believed that without this foundational knowledge, the perceived benefits and/or the relevance of the tool amongst practitioners would be reduced. Some practitioners expressed the view that the tool would be more readily used where a teacher and SLT work regularly together such as in a specialist setting.

#### Contextual considerations- inner setting

Inner setting factors relate to characteristics of the school that might influence implementation. Three core concepts related to this domain were identified: (a) engaged leadership (b) learning culture, and (c) implementation climate. See Table 5 for illustrative quotes in relation to inner setting factors.

**Table 5:** Illustrative quotes related to the inner setting.

Inner setting factors	Descriptive summary of findings	Illustrative quotes
**Leadership engagement**	Engaged leadership is critical to innovation in schools	“Leadership has changed in the school recently… and straight away progress is being made… I know for example the new management are supportive of teachers going on …the master’s for the special needs coordination and that kind of stuff… Changes in the structure at the moment has made it so much better already” (P10, subject teacher, post-primary)
	Distributed leadership where everyone proactively engages in change	“Staff actively engage everybody and make everybody feel welcome and that they have a voice, and that gives us energy for change as a group” (PI, deputy principal)
**A culture of learning and shared values**	When teaching staff are supported to upskill, when learning is valued and when there is sufficient time and space for reflective thinking and evaluation, innovation is possible.	“Yeah, anything that you want to try, anything new. . . he’s very supportive… So, because we’re both in special ed at the moment. …you know like doing this course, for example, that’s no problem. Same when C. did reading recovery, you know, it’s encouraged, actually, that’s why we are so up for trying new things… it is so important for change” (P7 class teacher, primary)
	Where there are shared collective values, change is possible	‘We all really value equity…so I think we are all very aware of what we want to achieve… that’s what drives us as a learning school” (P2 class teacher, primary)
**Implementation climate**	Top down, bureaucratic demands act as a barrier to leading change in schools	“We applied for additional SNA access in April, and we were granted nothing for 120 pages of an application…we reapplied a month later because we got a one page back to request more information… So, we reapplied in May or the start of June with 170 pages of an application. Like loads of information on the specific children. And that we also were given no extra allocation of SNA for that at that time. So, we reapplied at the start of September, and we heard nothing for 3 weeks and eventually I had to email. like when you are doing that all hours, what hope of me being able to lead on anything new happening” (P1, deputy principal& class teacher, primary)
	Enforced leadership roles are not conducive to innovation	‘During it (enforced time in acting school leadership role) you just keep going but afterwards when I took a step back and took time to reflect it definitely affected me, my happiness, my sense of worth like, as well, a little bit. I work very hard; I’ve done lots of additional courses. I really care about the kids. I care about my work, but the administrative work is just so frustrating and no time for anything new whatsoever.’ (P12, deputy principal, post-primary)

#### Engaged leadership

All participants spoke of the critical role of engaged leadership in implementing changes in classroom practice. When the principal of the school prioritises a new initiative, and directs time and resources to it, staff are more likely to implement innovative practices. Conversely, without engaged leadership, participants reported that initiatives were highly unlikely to become embedded into routine practice.

The importance of empowering others towards a model of distributed leadership and collective ownership of change was discussed by school leaders and practitioners alike. Practitioners were of the view that a school principal who listens to staff views and builds on their priorities for change can make school improvements happen. The importance of understanding the dynamics of change in schools on the part of school leaders was also discussed as important.

#### A learning culture

A learning culture was identified by all participants as critical for change. While many of the teachers interviewed had been supported by their school leader to complete additional qualifications and training, this was not considered sufficient to foster such a culture. Participants described the need for further learning opportunities both within their school setting, as well as with teachers from other schools. In one setting, where there was scheduled, protected peer learning time embedded in the school calendar, teachers reported feeling supported to try new methods. Views of the effectiveness of the outside speaker in creating a learning climate were equivocal. Some teachers expressed the view that such speakers brought useful knowledge, but others viewed the outside speaker as disempowering to teachers, acting as an impediment to them taking on leadership roles around their own professional learning.

The importance of having a collective vision and shared values as a school community was discussed by practitioners. One school leader noted that because all the staff were committed to redressing inequities of access to education, it helped sustain a series of practice changes in relation to inclusive pedagogy. One school leader gave practical examples in their own leadership practice where they had instigated changes to timetables and cycles of work to enable collaborative reflective sessions, which resulted in changes to classroom practice.

#### Implementation climate

All participants spoke of excessive administrative workloads and change fatigue as negatively impacting their absorptive capacity for change. From the perspective of school leaders, such demands were described as particularly challenging, leaving limited energy for innovative thinking. One school leader spoke of being ‘forced’ to take on a leadership role temporarily to fill a vacancy in school, describing the sense of isolation and burden of management responsibility during this time. Many practitioners and the school leaders interviewed described being at saturation point because of the administrative demands of their job. They reported that any innovation not fitting with existing work cycles and systems already in place in school were unlikely to succeed. One participant stated that just setting up the observation meeting and a follow up peer discussion for the pilot was challenging.

### Factors related to the outer setting

Outer setting factors include macro level influences on implementation of innovations in school. Two key factors were identified across the dataset related to the outer setting: (a) external policies and incentives and (b) partnerships and connections. See Table 6 for illustrative quotes in relation to outer setting factors.

**Table 6:** Illustrative quotes related to the outer setting.

Outer Setting	Descriptive summary of findings	Illustrative quotes
**External policies and incentives**	Department of Education as a driver of (unpleasant) change which must be complied with hinders innovation	‘But the other thing is we’re expecting a whole school inspection next week. And that’s another thing like. There’ll be loads of stuff to do after that. We have to sort the things they say, the department expects it… You know she (the inspector) is only going to give out about things. So, I’m like (sigh) a bit disillusioned.” (P1, deputy principal & class teacher, primary)
	Lack of clarity regarding professional roles and responsibilities in relation to SLCN and SEN	“Some subject teachers think it is not their business, it is for the SET to do work with the child, the SET is paid to do that work… and the department doesn’t really help with that giving money to new specialist roles or settings…I mean, I have seen withdrawal (from the classroom) for absolutely everything you can think of. . . I’d walk into meetings with large groups of people, and they would be saying “she’s doing this and she’s doing that” and then the SET will say “ok I’ll take her every Tuesday for 40 minutes and we’ll draw pictures and I’ll say that’s special education.” (P11, school principal, post-primary)
**Partnerships and connections**	A lack of partnership with local SLT services means schools and teachers go it alone	“…like in relation to then contacting external groups like the speech and language. That is very difficult. Sometimes you get work handed to you that has speech and language difficulties. But it generally tends to be basic and photocopied so I am none the wiser. I could do with the help from the SLT. . . . Imagine it would be great to see it. I’m sure the SLTs are sitting somewhere as well going God it would be great to go in into schools” (P7, class teacher, primary)
	The tool could enable partnerships of trust and to bridge differences across health and education	“…kind of like an ongoing relationship with teachers in terms of like checking in and that kind of thing, like when you work with someone ongoing you know you can build trust and that is important for SLTs and teachers you know because they are so different. Different backgrounds and different ways of thinking about things. So, this could be useful, but you would have to know each other first and trust each other.” (P3, speech and language therapist, special class)

#### External policies and incentives

Participants felt that schools are inundated with directives from the Department of Education, and that these directives negatively impact the potential for innovation in schools. School leaders discussed how these demands hindered their ability to lead change. School inspections were discussed by all participants as an unpleasant driver of change. Participants felt that inspection findings had to be complied with and prioritised over any school-driven initiatives.

Mixed messaging in education policy with regards inclusion and special education was also discussed by school leaders and practitioners as a potential barrier to implementing the tool. A lack of understanding of SEN models of support was also discussed by participants as a potential obstacle to implementation of the tool in some school settings.

#### Partnerships and connections

All participants discussed the lack of connectedness with their local SLT health services as a barrier to implementing the tool. Only one of the schools in the study had a regular visit from an SLT. Participants described being ‘left alone’ to deal with the SLCN of children, reporting limited knowledge of strategies they could use to meet these needs in the classroom. Teachers suggested that the tool could help support collaborative planning with the SLT in an ideal world but given the lack of SLT visits, they were of the view that such potential was unlikely to be realised.

### Factors related to the implementation process

This domain relates to the *processes* that would support use of the tool in the school setting. Two key factors were identified across the dataset related to the implementation process: (i) Planning, and (ii) Champions. See Table 7 for illustrative quotes in relation to implementation planning considerations.

**Table 7:** Illustrative quotes related to the process of implementation.

Process	Descriptive summary of findings	Illustrative quotes
**Planning**	Embedding use into the whole school evaluation process is critical to implementation	“…bringing it in under the school self-evaluation plan. Because if there’s already an established school self-evaluation team in the school. You’re not going in saying this is new where are you going to put it?” (P12, deputy principal, post-primary)
	Protected time needs to be scheduled schoolwide for collaborative reflection	“If it (protected time) was available that would definitely encourage people to do it because it’s the thought of doing it and then having to go back over it again yourself is something that might turn me off” (P2 class teacher, primary)
	Implementation timing is important	“I think the time of year introducing everything in a post primary school is so important. That’s what I would say August September is a good time when I’m introducing the needs especially of our first years and updating teachers on our needs of our second to sixth years” (P10, subject teacher, post-primary)
	Use of the tool needs to be regular, fitting with teaching cycles of work	“Take our block of teaching… we have we’ll say 8 weeks or nine weeks starting September October then a break and then another eight weeks teaching. Wouldn’t it be fabulous if this could be completed every nine weeks. To implement you know maybe different strategies I would say at least three to four times a year. Because once a year you forget you know” “Schools could complete this tool at the start of the year and review two months later. Don’t wait too long. Review it within a short space of time not at the end of the year. At the end of the year, we’re hanging. We’re gone you know” (P5, special education needs coordinator & deputy principal, post-primary)
	Implementation needs to be built into existing collaborative practices	“… you really do want to get to where you have the two teachers actively leading the class already to use the tool–setting up something extra even if it only takes 30 minutes can feel almost impossible some days” (P6, subject teacher, post-primary)
**Champions**	A champion in the school is needed to engage others in implementing the tool	“Yeah, it’s interesting to hear how like sometimes it can take one really motivated person to kind of just lead it a little bit and then you know the practice changes. Through that, rather than say or, there’s a new policy that that seems to be not enough in itself.” (P1, deputy principal & class teacher)

#### Planning

Practitioners and school leaders identified planning at a school level as essential if the tool were to be embedded into routine practice. Frequently discussed factors across both primary and post-primary school settings were timing, compatibility with existing processes and procedures, and regularity of use. Participants described the school year in terms of teaching blocks of eight weeks and advised that the tool should be planned to be used early in the first eight weeks of the school year. The importance of regular opportunities to review the tool was also highlighted. Leaving too long a gap of time between first using the tool and reviewing changes in practice would impact implementation. Participants agreed that the tool would be most compatible in school settings where co-teaching was already in place in the classroom. Schools and teachers who are unfamiliar with co-teaching would require more awareness building about the tool and would require more support to use it.

#### Champions

The role of the champion was also noted as important by participants. Where one motivated person gets behind a new idea in a school, participants felt they can be very effective in engaging others. Participants cited examples of how support from a known and trusted colleague had resulted in school changes being maintained. Without support, participants viewed it unlikely that new initiatives such as the CSCOT would be used routinely. One pair of participants noted that external facilitation had also been effective provided it is sustained, describing a project which was successful for the duration that an external facilitator was involved but which failed to be maintained once that individual moved on to another school.

## Discussion

We engaged stakeholders working in Irish schools to trial the use of the ‘Communication Friendly Classroom Tool’ (CSCOT), developed as part of the Better Communication Research Project in the UK. The tool was trialed by a diverse sample of practitioners, in a range of school settings. The aims of the study were to evaluate the acceptability, feasibility, and appropriateness of the tool, and to gain insights into the strategies required to support the adoption, implementation, and sustainment of the tool in Irish schools. An advisory panel of teachers and a further panel of children with SLCN were engaged as co-researchers to review the tool prior to trialing it, and to make recommendations based on the findings as to whether further research involving the tool was warranted.

Participants viewed the CSCOT as acceptable, suggesting several advantages to its use. The adaptability of the tool was noted as a positive intervention characteristic. In relation to content, the Language Learning Interaction, and the Language Learning Opportunity sections were deemed relevant by participants across both primary and post-primary settings, with some suggested additions. Consistent with the views of the teachers in the advisory panel, the Language Learning Environment was considered to have limited relevance for post-primary mainstream settings.

The tool was viewed as particularly compatible for use in classrooms where co-teaching is routine practice. Although potentially exposing, where used with a trusted colleague, participants noted that the tool could be validating of their practice. Benefits of the tool as a shared point of reference about communication and interaction in the classroom between SLTs and teachers were identified by the participants who trialed it. However, participants noted the lack of connectedness between schools and health services at the outer context level meant that such a benefit was unlikely to be realised.

There were several intervention-related factors which were not discussed by practitioners involved in trialing the tool. For example, the source of the intervention i.e., where it was developed and/or the evidence behind the tool were not a focus of interest. This is surprising given the emphasis on evidence-informed practice in health and education training and in continuing professional development of teachers and SLTs [69,70]. Participants did not discuss the cost of the tool as an influencing implementation factor. This finding is also unexpected as resource issues are frequently cited as a barrier to implementing new innovations in the context of public health and public education systems elsewhere [71].

At the practitioner level, consistent with previous studies, the importance of self-efficacy of teachers, and their values and beliefs (in this instance relation to inclusive education) were identified as influential factors in implementing the CSCOT [72–74]. The lack of knowledge about the continuum of support model for SEN and about SLCN identified in this study has been documented in Ireland and elsewhere previously [4,9]. If teachers do not understand their role in ensuring the delivery of universal level SLCN supports, then they may not see a need for the tool. Gaps in knowledge about the impact of SLCN on learning may also negatively impact tension for change. Tension for change, or the degree to which stakeholders perceive the current situation as needing to be improved, is known to influence the likelihood of implementing innovations [74,75].

Practitioners were of the view that school settings vary in relation to their training needs with regards to understanding the continuum of support, the role of the class or subject teacher in supporting SLCN, and the use of reflective tools. These finding suggest that a dynamic training approach as part of an implementation strategy in a future pilot study is indicated. Such an approach includes methods of ascertaining a baseline in relation to such knowledge and motivation and allows for a more responsive and feasible educational strategy [76–78]. Given the reflective nature of CSCOT, explicit teaching of evidence-based problem-solving models such as a Plan-Do-Study-Act cycle, and facilitation techniques such as coach and consult may enable modelling and simulation use of the tool [66]. In the context of current SLT service delivery and school support models, such a training strategy could be co-designed by an SLT and school staff, as ownership of training can increase motivation [79,80].

Participants spoke of the importance of facilitators in engaging and supporting others in change in the school context. Given that there are designated roles to lead on implementation of schoolwide SEN supports, and that engaging and supporting school staff in the implementation of SLCN programmes is a core component of the role of visiting SLTs, there is potential capacity for both an internal school champion and an external SLT facilitator to support implementation of the tool. Previous research related to SLT work in schools suggests that where these professionals have a clear scope and role, many of the barriers to relationship-building and leading on change can be overcome [81]. As partnerships between academia and schools have been shown to be effective in sustaining changes in classroom practice, both internal and external facilitators of CSCOT would benefit from opportunities for cross-agency professional learning networks and/or engagement with communities of practice at a regional level, to support implementation of the tool across schools [82–84].

Whilst adaptability and fit were positive features of the CSCOT, there was strong agreement across all stakeholders that integrating the CSCOT into schoolwide (inner setting) systems would be essential to support implementation of the tool. This finding is consistent with previous studies which show that inadequate attention to system influences can undermine even the most well-resourced and thoughtful implementation strategies [31,32]. It was proposed by stakeholders that the tool could be integrated effectively into the school self-evaluation process, and school provision mapping processes undertaken in Irish schools [85,86]. The reflective nature of the tool was perceived to fit well with such data-informed decision-making approaches to action planning. Integrating the tool into these processes would address many of the issues raised related to planning of the use of the tool in terms of teaching cycles of work in the school context.

Consistent with implementation studies in school contexts internationally, the essential role of the school leader in promoting a learning climate, and capacity for change is highlighted [87–90]. Where the school leader signals their commitment to an innovation, and ensures it is integrated into inner context systems, participants were of the view that new practices can be sustained in Irish classrooms. It is noteworthy that the school leaders in this study view their own capacity to lead on innovation as being negatively impacted by the bureaucratic demands of the Department of Education. Nonetheless, individuals in these roles have described successful methods of leading on change despite the administrative demands of the outer setting. Findings are clear that school leaders will be critical partners in the planning, and delivery of any future implementation universal level SLCN intervention in Irish schools. A summary of the views of the tool from those who trialed it were shared with the two advisory panels. Both teachers and children agreed that the tool should be further tested in Irish schools. Post-primary teachers suggetsed the need for further consideration of the language of the tool for their setting, and the removal of the Language Learning Environment section prior to testing.

## Strengths and limitations

A careful sampling strategy enabled rich descriptive data to be captured in relation to the intervention characteristics, and implementation of the CSCOT tool in a diverse sample of Irish schools. Whilst we did succeed in sampling both primary and secondary schools, only one post-primary school in the sample was designated as DEIS. Given we know that speech, language, and communication needs are disproportionately represented in populations of high socio-economic need, including an additional DEIS primary school would have provided further insights into the feasibility, relevance, and appropriateness of the CSCOT related to this specific setting.

Because we sampled by school, and only one school recruited was receiving regular support from an SLT at the time of the study, it was not possible to explore views of the CSCOT as a tool to support collaboration between an SLT and class teacher as was planned. Including the perspectives of SLTs in the next stages of the research will be essential given these professionals are responsible for conducting much of the practice of implementation of SLCN interventions in schools.

In this exploratory study, practitioners were given the autonomy to choose which lesson, which age ranges, and with whom they trialed the tool. As the next stages of the research would be experimental in nature, it will be necessary to either specify when the tool is best used, or to record the impact of these differences in use across sites.

Finally, it is important to note that the qualitative inquiry was undertaken during the COVID pandemic, an unprecedented time of change in schools. This may have influenced practitioners and school leaders’ views in relation to absorptive capacity for change.

## Conclusions

Successful implementation of universal level interventions in school can ensure the participation and achievement of children and young people with speech, language, and communication needs. Findings from this exploratory study with stakeholders from a purposive sample of schools has provided important insights into the acceptability, feasibility, and acceptability of a tool aimed at optimising communication and interaction in the Irish school context.

The critical role of school leaders in building capacity for change in Irish schools is highlighted. Useful suggestions with regards to contextual integration of the tool were generated. Differences at a school level in relation to implementation needs were noted, suggesting the need for an adaptive study design in a future pilot study. Data from the study has the potential to guide the co-development of a feasible implementation strategy with stakeholders. The importance of early-stage exploration to guide implementation research planning in schools is highlighted.

## Supporting information

S1 File The communication supporting classroom observation tool. (PDF)

S2 File Background questionnaire and topic guides. (PDF)
