# Supplementary material for: Exploring the acceptability, feasibility, and appropriateness of a communication-friendly classroom tool for use in Irish schools: A qualitative inquiry
Source: PLoS One. 2023 Jun 22;18(6):e0287471. doi: 10.1371/journal.pone.0287471 (PMC10286983; doi:10.1371/journal.pone.0287471)
Supplement: S1 File — (PDF) [file pone.0287471.s001.pdf]

2012
BETTER COMMUNICATION RESEARCH PROGRAMME

Developed by CsC Team:
Dockrell, J. E., Bakopoulou, I., Law, J., Spencer, S. & Lindsay, G.

Communication Supporting Classroom Observation Tool

The Communication Trust
Every child understood

2012
BETTER COMMUNICATION RESEARCH PROGRAMME

School ....

Date .... Start time .... Finish time ....

Completed by .... Class .... No of pupils ....

Overview of the Communication Supporting Classrooms Observation Tool

This tool was developed as part of the Better Communication Research Programme (BCRP) in 2012. The BCRP was a 3 year research programme that was part of the Government's response to the 2008 Bercow Review of provision of services for children with speech, language and communication needs (SLCN).

The Communication Trust are supporting the BCRP to share their findings.

Information about the tool:

- The observation tool is designed to be used in an observation of a classroom or a learning space by someone other than the adult working with the children.
- The observation tool can be used in Reception, Year 1 and Year 2 classrooms and other Early years learning spaces.
- The average length of time necessary to collect a representative sample of behaviour is approximately one hour. Some of the items of the first dimension (Language Learning Environment) can be done during break time or prior to the start of the school day.
- It is recommended that the observation takes place during a regular classroom session (usually a morning session starting with the class register).
- The language learning dimensions are recorded as either present or absent. For some items, there is a record of a Language Learning Opportunity being 'Present' and being 'Used during the Observation'.
- For the dimensions of 'Language Learning Opportunities' and 'Language Learning Interactions', each different occurrence is recorded up to a maximum of 5 times during the observation period. Each recorded observation is a new/ different occurrence of the behaviour/ activity.
- There is space when recording language learning interactions to note which staff use specific ways of talking with the children.

The tool is designed to profile the oral language environment of the classroom. It is not expected that all items will appear on all observations.

The Communication Trust
Every child understood

Language Learning Environment

This dimension involves the physical environment and learning context

		Not Seen	Observed	Comments
1	The classroom is organised to emphasise open space.			
2	Learning areas are clearly defined throughout the classroom.			
3	Learning areas are clearly labelled with pictures/words throughout the classroom.			
4	Space for privacy/quiet areas where children can retreat to have ‘down time’ or engage in smaller group activities. These areas are less visually distracting.			
5	Children’s own work is displayed and labelled appropriately.			
6	Some classroom displays include items that invite comments from children.			
7	Book specific areas are available.			
8	Literacy specific areas are available.			
9	Background noise levels are managed consistently throughout the observation, and children and adults are able to hear one another with ease.			
10	Transition times are managed effectively, so that noise levels are not excessive and children know what to expect next.			

11	There is good light.				
12	The majority of learning resources and materials are labelled with pictures/words.				
13	Resources that are available for free play are easily reached by the children or easily within their line of vision.				
14	An appropriate range of books is available in the book area (e.g, traditional stories, bilingual/dual language books and a variety of genres and books related to children's own experiences).				
15	Non-fiction books, books on specific topics or interests of the children are also available in other learning areas.				
16	Outdoor play (if available) includes imaginative role play.				
17	Good quality toys, small world objects and real/natural resources are available.		Present:	Used:	
18	Musical instruments and noise makers are available.		Present:	Used:	
19	Role play area is available.		Present:	Used:	
**Total Score**	**/19** **Notes:**				

**Language Learning Opportunities**<br>This dimension involves the structured opportunities that are present in the classroom to support language development								
		**Not Seen**	**Observed (5 times)**					**Comments**
1	Small group work facilitated by an adult takes place.							
2	Children have opportunities to engage in interactive book reading facilitated by an adult (for example: asking predictive questions, joining in with repetitions, story packs etc.).							
3	Children have opportunities to engage in structured conversations with teachers and other adults.							
4	Children have opportunities to engage in structured conversations with peers (Talking partners).							
5	Attempts are made to actively include all children in small group activities.							
**Total Score**	**/25** **Notes:**							

Language Learning Interactions

This dimension involves the ways in which adults in the setting talk **with** children.

		Not Seen	Observed					Observed by all staff in classroom	Comments
1	Adults use children's name, draw attention of children.								
2	Adults get down to the child's level when interacting with them.								
3	Natural gestures and some key word signing are used in interactions with children.								
4	Adults use symbols, pictures and props (real objects) to reinforce language.								
5	Pacing: Adult uses a slow pace during conversation; give children plenty of time to respond and take turns in interacting with them.								
6	Pausing: Adult pauses expectantly and frequently during interactions with children to encourage their turn-taking and active participation.								
7	Confirming: Adult responds to the majority of child utterances by confirming understanding of the child's intentions. Adult does not ignore child's communicative bids.								

8	Imitating: Adult imitates and repeats what child says more or less exactly.								
9	Commenting: Adult comments on what is happening or what children are doing at that time.								
10	Extending: Adult repeats what child says and adds a small amount of syntactic or semantic information.								
11	Labelling: Adult provides the labels for familiar and unfamiliar actions, objects, or abstractions (e.g. feelings).								
12	Adult encourages children to use new words in their own talking.								
13	Open questioning: Adult asks open-ended questions that extend children’s thinking (what, where, when, how & why questions).								
14	Scripting: Adult provides a routine to the child for representing an activity (e.g. First, you go up to the counter. Then you say “I want milk..”) and engages the child in known routines (e.g. “Now it is time for circle time. What do we do first?”).								
15	Adult provides children with choices (for example: “Would you like to read a story or play on the computer?”).								

16	Adult uses contrasts that highlight differences in lexical items and in syntactic structures.								
17	Adult models language that the children are not producing yet.								
18	Turn-taking is encouraged.								
19	Children’s listening skills are praised.								
20	Children’s non-verbal communication is praised.								
**Total Score**	**/100** **Notes:**								

2012
BETTER
COMMUNICATION
RESEARCH
PROGRAMME

Guidance on completing Communication Supporting Classrooms Observation Tool

On the following pages you will find guidance on how to complete the tool, which includes examples of what you may observe.

You will also find references which demonstrates the evidence used to develop the tool.

For more information please go to **www2.warwick.ac.uk/fac/soc/cedar/better**

The Communication Trust
Every child understood

Language Learning Environment

This dimension involves the physical environment and learning context

	Examples	Notes
The classroom is organised to emphasise open space.[1,4,6]		
Learning areas are clearly defined throughout the classroom.[1, 2,3,4,5,6,7,8,12]	Different learning areas, such as small world play, reading corner, maths area, construction, topic table, computer area are available within the classroom.	
Learning areas are clearly labelled with pictures/words throughout the classroom.[1, 2,3,4,5,6,7,8,12]	Symbols and pictures are used to label different areas, such as the kitchen and book areas.	
There is space for privacy or quiet areas where children can retreat to have 'down time' or	There is a big tent for children to go into with a book.	This item is specifically for quiet spaces. Classrooms may have spaces such as a house.
Children's own work is displayed and labelled appropriately.[5,6,7,8]	Self-portraits with labels and descriptions.<br>Children's drawings, potato prints.	
Some classroom displays include items that invite comments from children.[5,6,7,8]	"Can you order your numbers here?"<br>"How much did you enjoy our trip to the zoo?"<br>Children are encouraged to rate the trip using stars.	This item refers to displays which have space for children to contribute.
Book specific areas are available.[1, 3,4,5,6,7,8]	Book displays, shelves within easy reach.	

Literacy specific areas are available.[1, 3,4,5,6,7,8]	Desks with paper, whiteboards, pens and books to practise spelling, handwriting or reading.	Literacy specific areas may include materials for writing or practicing handwriting.
Background noise levels are managed consistently throughout the observation, and children and adults are able to hear one another with ease.[4,6,9,10,11]	Noise levels are managed well throughout the observation.<br>Soft music playing in the background during free play.	
Transition times are managed effectively, so that noise levels are not excessive and children know what to expect next.[4,5,7,9,10,11]	The adult rings a bell and all children stop and put both hands in the air and wait for instructions. A tambourine is used to signal the children have to wait and listen for the next instruction.	
There is good light.[4,5,6,8,12]		
The majority of learning resources and materials are labelled with pictures/ words.[4,5,6,7,13]		
Resources that are available for free play are easily reached by the children or easily within their line of vision.[4,5,6,7,8]	Blocks, play dough, toy animals, number lines within easy reach.	
An appropriate range of books are available in the book area (for example, traditional stories, bilingual/dual language books and a variety of genres and books related to children's own experiences).[13]		

Non-fiction books, books on specific topics or interests of the children are also available in other learning areas.[13]	Books on dinosaurs.<br>Books on transportation.<br>Space and the universe books and props.	
Outdoor play (if available) includes imaginative role play.[7,8,37]	Children dressed up as construction workers (high vis jackets and hard hats) for break outside.<br>Home corner available outdoors.	
Good quality toys, small world objects and real/natural resources are available. [1, 2,4,5,6,7,8,37]	Zoo toys, shells, pebbles, seeds.<br>Castle set and toys related to topic.	
Musical instruments and noise makers are available. [1,2,4,5,6,7,8,37]	Adult uses the tambourine to get children's attention.<br>Adult plays the guitar during story time.<br>Children take turns to use the wooden flutes while the adult reads a story.<br>Concept of pitch is explored using bells.	
Role play area is available. [1,2,4,5,6,7,8,37]	Kitchen area.<br>Puppets and soft animals used for imaginary play.<br>In the kitchen area there are different outfits for children to wear.<br>Castle costumes in the class (e.g. knight and princess).	

**Language Learning Opportunities**<br>This dimension involves the structure opportunities that are present in the setting to support language development.		
	**Examples**	**Notes**
Small group work facilitated by an adult takes place. [16, 17, 18, 19,58]	Phonics groups (children grouped by ability).<br>Letter-sound matching activity within small groups.<br>Counting practice group.<br>Children complete spelling tasks, sitting on different tables according to ability (labelled by different animal names) with adult support.	
Children have opportunities to engage in interactive book reading facilitated by an adult (for example: asking predictive questions, joining in with repetitions, story packs etc.). [14,5,17,19,20,21,25,26,27,28,29,30,31,32,58]	Teacher reads two books brought in by a child from home. During the reading she asks two questions (“Why would Mr Stick be scared of a dog?” “What are baby butterflies?”).	
Children have opportunities to engage in structured conversations with teachers and other adults.[19,20,21,22,23,24,33,34,58]	Adult sits at the free play tables and answer children’s questions, comments on their activities, asks questions and follows up conversation.<br>Children approach adult with news about family, adult asks questions and comments, relating to background knowledge of prior events.<br>Show and Tell carpet time includes questions that require from the child to provide more information on the object.	Conversations are structured by following the child’s lead, attending to the child and talking about what the child is doing or is interested in with an emphasis on taking turns.

Children have opportunities to engage in structured conversations with peers (Talking partners). [35,36,58]	Children discuss a topic with the child sitting next to them during carpet time and give a joint answer to the whole-group.<br>Children work in pairs – one describes a geographical shape while the other guesses which shape they are thinking of.	Children are given prompts and support by adults to engage in a specific conversation about the current topic.
Attempts are made to actively include all children in small group activities. [23,37,58,62]	Less talkative children are identified by adults, who invite them to sit on their knee to have a conversation.<br>Additional modification of language is used by adults to include less-talkative children in whole-class discussions.	

Language Learning Interactions

This dimension involves the ways in which adults in the setting talk with children

	Examples	Notes
Adults use child's name, draw attention of the child. [1,38,39,40,41,44,45,46,47]	Adult says the name of each child before giving them a counting task (e.g. Sarah – 3+4!)<br>During greetings at the start of the day.<br>Adult uses the child's name to get their attention ***before*** asking them a specific question during 'show and tell' session.	If an adult does this repetitively during one activity (e.g. a counting task), but does not use this strategy during the rest of the session, you may wish to count the incidence as 'once' (rather than counting the individual occurrences within the one task).
Adults get down to the child's level when interacting with them. [1,38,39,40,41,44,45,46,47]	Adult sits on the carpet with the children to complete a maths activity.<br>Adult sits on small chairs designed for children during free activity time.	
Natural gestures and some key word signing are used in interactions with children. [39,40,41,42,43,44,45,46,47]	Thumbs up.<br>Use a gesture for 'big' (tower).<br>Use the 'where' Makaton sign.<br>Gestured when saying 'I can see a long way'.<br>Fingers to signal 3 hats.<br>Five minutes (hand gesture for 5).<br>Knock it over (gesture for knock!).<br>When instructing in an ICT lesson, teachers use gestures for up/down/left/right/high/low.<br>Iconic gestures are used, e.g. gesture for 'cliff' (in discussion of what an edge is in maths lesson).	

Adults use symbols, pictures and props (real objects) to reinforce language.[1]	Visual timetable displayed, with a focus on a child who has recently moved to the area from abroad and a child with ASD.<br>Pointing at pictures when reading a story.<br>Holding a wooden train toy and referring to it when talking about transportation.	
Pacing: Adults use a slow pace during conversation; give children plenty of time to respond and take turns in interacting with them.[1,19,21,34,39,40,41,44,45,46,47]	When explaining how to log on to the computers, the adult takes lots of pauses and talks slowly.	
Pausing: Adults pause expectantly and frequently during interactions with children to encourage their turn-taking and active participation.[1,19,21,44,45,46,47]	Counting activity "– 2, 4, 6…!"<br>**Adult:** "How do we call this? It's a… pancake!"<br>**Adult:** "What day is it today, do you know?…. It was Monday yesterday so it's… Today is - Tuesday!"	
Confirming: Adults respond to the majority of child utterances by confirming understanding of the child's intentions. Adults do not ignore child's communicative bids. [1,19,44,45,46,47,48]	Adult confirms if answer to counting was correct?<br>**Child:** "My grandmother has rabbits in her garden". **Adult:** "That sounds interesting, tell me about the rabbits later"<br>**Child:** "Look Miss!" **Adult:** "Oh look what you've done! He's made a car!"<br>**Child:** "Miss, look at my star!" **Adult:** "Oh wow… this is a big bright star!"	

Imitating: Adults imitate and repeat what child says more or less exactly. [1,19,44,45,46,47,48]	**Child:** “It is my sister’s birthday on Saturday”. **Adult:** “Is it really her birthday? How exciting”.<br>**Child:** “Miss look at my tower”. **Adult:** “Oh wow… look at your tower!”	
Commenting: Adults comment on what is happening or what children are doing at that time. [1,19,44,45,46,47,49,50, 51]	**Adult:** “Charlie, that’s a great design”.<br>**Adult:** “A spider! Your favourite animal!”<br>**Adult:** “I like the way Alfie and Tiana put all the blocks together to build a really tall tower.”<br>**Adult:** “I can see what you’re doing, you’re trying to copy.”	In order to be scored, the adult’s comment should be directed at the child(ren) and be about the immediate situation.
Extending: Adults repeat what child says and add a small amount of syntactic or semantic information. [1,19,44,45,46,47,48,49,50, 51]	**Child:** “Because Cinderella was scared of her sisters”.<br>**Adult:** “That’s right. Cinderella was scared of her two horrible sisters”.<br>**Child:** “My mummy brought me here”. **Adult:** “Your mummy’s brought you here has she? She’s seen you to the gate. Here she is!”<br>**Child:** “Chimney house”. **Adult:** “Chimney that’s like the one we saw when we went on our walk”.<br>**Child:** “Look at my dress”. **Adult:** “It’s a very beautiful summer dress”.	

Labelling: Adults provide the labels for familiar and unfamiliar actions, objects, or abstractions (e.g. feelings).[54,55,56,58,59,60]	**Child:** "I need to be careful." **Adult:** "That's right. You need to be precise"<br>**Adult:** "What's another word for punch? (Pause) Starts with "h".<br>**Adult:** "When someone doesn't feel excited in a nice way, we say they feel...(pause) upset".<br>The adult describes the word octagon in relation to an octopus.<br>Introduces the words pentagon, cylinder, cuboids, and cone.	
Adults encourage children to use new words in their own talking. [54,55,56,58,59,60]	**Adult:** "What's another word for that...?"<br>**Adult:** "Submarine (what did we call that one again?)"<br>**Child:** "They rhyme". **Adult:** "That's right. We learnt about rhyming in the morning".	
Open questioning: Adults ask open-ended questions that extend children's thinking (what, where, when, how & why questions). [1,19,44,45,46,47,52,53,57,58]	"How does it change from one to another?"<br>"What did you like about the way Tiara read the story?"<br>"What do you know about a giant's house?"<br>"Why do you think they might be hot?"<br>"How's it different to a square?"<br>"And what's this book about?"	

Scripting: Adults provide a verbal routine to the child for representing an activity (e.g. First, you go up to the counter. Then you say "I want milk..") and engage the child in known routines (e.g. "Now it is time for circle time. What do we do first?"). [1,19,44,45,46,47,58]	When we do a book review, we say "I gave Cinderella three stars because..."	Scripts provide children with accurate verbal information about those situations or activities they may encounter. The situation or activity is described in detail providing the child with a script of what to say or do, what might be expected of them and why. This item should not be scored if the adult just gives directions (e.g. **Adult:** "Now go to your tables and start the task").
Adults provide children with choices (for example: "Would you like to read a story or play on the computer?"). [1]	"Do you want to go outside or go on the computer?"<br>"Do you want to show us a magic trick or tell us about last night (in Show and Tell)?"	
Adults use contrasts that highlight differences in lexical items and in syntactic structures.[51,54,55,56,58,59,60,61]	Amphibian crafts versus hovercrafts!<br>Smaller v smallest.<br>"That's not just a car, it's like a minibus!"<br>"Hammer doesn't start with d, that would be dammer."<br>The adult explains to the children the meaning of the words content and index.<br>Face versus Side<br>Sophia versus spear versus sphere!<br>Discusses a face of a circle versus a face of a 2d shape in maths.	

Adults model language that the children are not producing yet.[58]	What are the properties of the shape?	Adults may use a word or sentence structure which you would not expect of a child in key stage 1. In order to score on this item, consider if the adult is using language which is within the child's zone of proximal development – e.g. is the language use helping develop children's language skills? Or is it too complex to be accessed by children of this age range (in which case, do not score a point)?
Turn-taking is encouraged. [1,62]	**Adult:** "We are working as a team - doing it all together. Now it's my turn, then it's Amber's turn."<br>**Adult:** "Let's take it in turns to think of a word to describe the monster."	
Children's listening skills are praised. [1,62]	**Adult:** "That's very good listening."<br>**Adult:** "I can tell you are listening to me by the way you all look at me when I explain the task. Great listening!"	This item is scored if listening is explicitly praised. It does not include praise for being quiet (e.g. "this class is really quiet – good work" would not be scored) or discipline for poor listening (e.g. "I wish there was more listening going on in here today!"). You may wish to note any positive strategies that the adults use to encourage good listening.
Children's non-verbal communication is praised. [1,62]	**Adults:** "I like the way you look at me when I explain the exercise. It makes me think you are really listening at me."	

References

1. Justice, L.M. (2004). Creating Language-Rich Preschool Classroom Environments. *Teaching Exceptional Children,* 36-44.
2. Justice, L. M., MCGinty, A., Guo, Y., & Moore, D. (2009). Implementation of responsiveness to intervention in early education settings. *Seminars in Speech and Language, 30*(2), 59-74.
3. Bond, M. A., & Wasik, B. A. (2009). Conversation Stations: Promoting Language Development in Young Children. *Early Childhood Educational Journal, 36,* 467-473.
4. Siraj-Blatchford, I., Sylva, K., Muttock, S., Gilden, R., & Bell, D. (2002). Researching effective pedagogy in the *early years. London: DFES.*
5. Harms, T., Clifford, R. M., & Cryer, D. (1996). *Early Childhood Environment Rating Scale - Revised (ECERS-R).* London: Teachers College Press.
6. Sylva, K, Siraj-Blatchford, I., Taggart, B. (2006). *Assessing Quality in the Early Years: Early Childhood Environmental Rating Scale – Extension (ECERS-E).* Stoke-on Trent, UK and Sterling, USA: Trentham Books.
7. I CAN (2008). I CAN Early Talk: A Supportive Service for Children's Communication. Accreditation Standards.
8. Communication Trust (2008). The Speech, Language and Communication Framework. www.talkingpoint.org.uk/sclf
9. Dockrell, J. E., & Shield, B. M. (2004). Children's perception of their acoustic environment at home and at school. *Journal of the Acoustical Society of America, 115,* 2964-2973.
10. Shields, B.M., & Dockrell, J.E. (2008). The effects of environmental and classroom noise on the academic attainments of primary school children. *Journal of the Acoustical Society of America, 123*(1), 133-144.
11. Dockrell, J. E., & Shield, B. M. (2006). Acoustical barriers in classrooms: the impact of noise on performance in the classroom. *British Educational Research Journal, 32*(3), 509-525.
12. Building Bulletin 87, BB 87, Guidelines for Environmental Design in Schools (DCSF) http://teachernet.gov.uk/energy
13. Dowhower, S. L., & Beagle, K. G. (1998). The print environment in kindergartens: A study of conventional and holistic teachers and their classrooms in three settings. *Reading Research and Instruction, 37*(3), 161-190.
14. Justice, L.M., Kaderavek, J.N., Fan, X., Sofka, A., & Hunt, A. (2009). Accelerating Preschoolers' Early Literacy Development Through Classroom Based Teacher-Child Storybook Reading and Explicit Print Referencing. *Language Speech and Hearing Services in Schools, 40*(1), 67-85.
15. Mol, S., Bus, A., & de Jong, M. (2009). Interactive book reading in early education: A tool to stimulate print knowledge as well as oral language. *Review of Educational Research, 79,* 979–1007.
16. Wasik, B. A. (2008). When fewer is more: Small groups in early childhood classrooms. *Early Childhood Education Journal, 35,* 515-521.
17. Morrow, L. M., & Smith, J. K. (1990). The effects of group size on interactive storybook reading. *Reading Research Quarterly, 25,* 213-231.
18. Turnbull, K. P., Anthony, A. B., Justice, L., & Bowles, R. (2009). Preschoolers' exposure to language stimulation in classrooms serving at-risk children: The contribution of group size and activity context. *Early Education and Development, 20*(1), 53-79.
19. Dockrell, J. E., Stuart, M., & King, D. (2010). Supporting early oral language skills for English language learners in inner city preschool provision. *British Journal of Educational Psychology, 80*(4), 497-515.
20. Saunders, W. M., & Goldenberg, C. (1999). Effects of instructional conversations and literature logs on limited- and fluent-English-proficient students' story comprehension and thematic understanding. *Elementary School Journal, 99*(4), 277–301.
21. Carlo, M. S., August, D., McLaughlin, B., Snow, C. E., Dressler, C., Lippman, D. N., White, C. E. (2004). Closing the gap: Addressing the vocabulary needs of English-language learners in bilingual and mainstream classrooms. *Reading Research Quarterly, 39,* 188–215.
22. Bickford-Smith, A., Wijayatilake, L., & Woods, G. (2005). Evaluating the Effectiveness of an Early Years Language Intervention. *Educational Psychology in Practice, 21*(3), 161-173.
23. Best, W., Melvin, D., & Williams, S. (1993). The effectiveness of communication groups in day nurseries. *European Journal of Disordered Communication, 28,* 187–212.
24. NICHD Early Child Care Research Network (2000). The relation of child care to cognitive and language development. *Child Development, 71,* 960–980.
25. Collins, M. (2010). ELL preschoolers' English vocabulary acquisition from story book reading. *Early Childhood Research Quarterly, 25,* 84–97.
26. Hargrave, A. C., & Sénéchal, M. (2000). A book reading intervention with preschool children who have limited vocabularies: The benefits of regular reading and dialogic reading. *Early Childhood Research Quarterly, 15,* 75–90.
27. Koshinen, P. S., Blum, I. H., Bisson, S. A., Phillips, S. M., Creamer, T. S., & Baker, T. K. (2000). Book access, shared reading, and audio models: The effects of supporting the literacy learning of linguistically diverse students in school and at home. *Journal of Educational Psychology, 92*(1), 23-36.
28. Dickinson, D. K. (2001). Book reading in preschool classrooms: Is recommended practice common? In D. K. Dickinson & P. O. Tabors (Eds.), *Beginning literacy with language: Young children learning at home and school* (pp. 175-203). Baltimore: Brookes Publishing Company.
29. Ezell, H.K., & Justice, L. M. (2005). *Shared storybook reading: Building young children's language and emergent literacy skills.* Baltimore, MD: Paul H. Brookes.
30. Justice, L.M., & Ezell, H.K. (2002). Use of storybook reading to increase print awareness in at-risk children. *American Journal of Speech-Language Pathology*, 11(1), 17-29.
31. Justice, L. M., Meier, J., & Walpole, S. (2005). *Learning new words from storybooks: Findings from an intervention with at-risk kindergarteners*. Language, Speech, and Hearing Services in Schools, 36, 17-32.
32. Justice, L. M., & Pence, K. (2005). *Scaffolding with storybooks: A guide for enhancing young children's language and literacy achievement.* Newark, DE: International Reading Association.
33. Huttenlocher, J., Vasilyeva, M., Cymerman, E., & Levine, S. C. (2002). Language input at home and at school: Relation to syntax. *Cognitive Psychology*, 45, 337–374.
34. Justice, L. M., Mashburn, A. J., Hamre, B. K., & Pianta, R. C. (2008). Quality of language and literacy instruction in preschool classrooms serving at-risk pupils. *Early Childhood Research Quarterly*, 23(1), 51-68.
35. Mashburn, A. J., Justice, L. M., Downer, J. T., & Pianta, R. C. (2009). Peer effects on children's language achievement during pre-kindergarten. *Child Development*, 80(3), 686-702.
36. Justice, L.M., Petscher, Y., Schatschneider, C., & Mashburn, A. (2011). Peer effects in Preschool Classrooms: Is Children's Language Growth Associated with Their Classmates' Skills? *Child Development*, 82(6), 1768-1777.
37. Smith, M. W., & Dickinson, D.K. (1994). Describing oral language opportunities and environments in Head Start and other preschool classrooms. *Early Childhood Research Quarterly*, 9, 345-366.
38. Silverman, R., & Hines, S. (2009). The effects of multimedia-enhanced instruction on the vocabulary of English-language learners and non-English language learners in pre-kindergarten through second grade. *Journal of Educational Psychology*, 101, 305–314.
39. Gersten, R., & Baker, S. (2000). What do we know about effective instructional practices for English language learners? *Exceptional Children*, 66, 453–470.
40. Justice, L. M., Mashburn, A., Pence, K. L., & Wiggins, A. (2008). Experimental evaluation of a preschool language curriculum: Influence on children's expressive language skills. *Journal of Speech Language and Hearing Research*, 51(4), 983-1001.
41. Girolametto, L., Weitzman, E., van Lieshout, R., & Duff, D. (2000). Directiveness in teachers' language input to toddlers and preschoolers in day care. *Journal of Speech, Language, and Hearing Research*, 43, 1101–1114.
42. Launonen, K. (1996). Enhancing communication skills of children with Down syndrome: Early use of manual signs. In S. von Tetzchner, & M. H. Jensen (Eds.), *Augmentative and alternative communication: European perspectives.* London: Whurr.
43. Remington, B., & Clarke, S. (1996). Alternative and augmentative systems of communication for children with Down syndrome. In J. Rondal, J. Perera, L. Nadel, & A. Comblain (Eds.), Down syndrome: *Psychological, psychobiological and socio-educational perspectives.* London: Whurr.

44. Girolametto, L., & Weitzman, E. (2002). Responsiveness of child care providers in interactions with toddlers and pre-schoolers. *Language, Speech and Hearing Services in Schools*, 33, 268-281.

45. Cabell, S.Q., Justice, L.M., Piasta, S.B., Curenton, S.M., Wiggins, A., Turnbull, K.P., & Petscher, Y. (2011). The impact of teacher responsivity education on preschoolers' language and literacy skills. *American Journal of Speech-Language Pathology*, 20(4), 315-330.

46. Girolametto, L., Weitzman, E., & Greenberg, J. (2006). Facilitating language skills – In-service education for early childhood educators and preschool teachers. *Infants and Young Children*, 19(1),36-49.

47. Girolametto, L., Weitzman, E., & Greenberg, J. (2003). Training day care staff to facilitate children's language. *American Journal of Speech-Language Pathology*, 12(3), 299-311.

48. Tsybina, I., Girolametto, L., Weitzman, E., & Greenberg, J. (2006). Recasts used with preschoolers' learning English as their second language. *Early Childhood Education Journal*, 34, 177–185.

49. Vasilyeva, M., Huttenlocher, J., & Waterfall, H. (2006). Effects of language intervention on syntactic skill levels in preschoolers. *Developmental Psychology*, 42, 164–174.

50. Peterson, C., Jesso, B., & McCabe, A. (1999). Encouraging narratives in preschoolers: An intervention study. *Journal of Child Language*, 26, 49–67.

51. McCathren, R. B., Yoder, P. J., & Warren, S. F. (1995). The role of directives in early language intervention. *Journal of Early Intervention*, 19, 91-101.

52. Massey, S. L., Pence, K. L., Justice, L. M., & Bowles, R. P. (2008). Educators' use of cognitively challenging questions in economically disadvantaged preschool classroom. *Early Education and Development*, 19(2), 340-360.

53. Zucker, T.A., Justice, L.M., Piasta, S.B., & Kaderavek, J.N. (2010). Preschool teachers' literal and inferential questions and children's responses during whole-class shared reading. *Early Childhood Research Quarterly*, 25(1), 65-83.

54. Childers, J. B., & Tomasello, M. (2002). Two-year-olds learn novel nouns, verbs, and conventional actions from massed or distributed exposures. *Developmental Psychology*, 38, 967-978.

55. Wasik, B. A. (2006). Building vocabulary one word at a time. *Young Children*, 61(6), 70-78.

56. Pearson, B. Z., Fernandez, S. C., Lewedeg, V., & Oller, D. K. (1997). The relation of input factors to lexical learning by bilingual infants. *Applied Psycholinguistics*, 18, 41–58.

57. De Rivera, C., Girolametto, L., Greenberg, J., & Weitzman, E. (2005). Children's responses to educators' questions in day care play groups. *American Journal of Speech-Language Pathology*, 14(1), 14-26.

58. Chapman, R. S. (2000). Children's language learning: An interactionist perspective. *Journal of Child Psychology and Psychiatry*, 41, 33–54.

59. McKeown, M. G., Beck, I. L., Omanson, R. C., & Perfetti, C. A. (1983). The effects of long-term vocabulary instruction on reading comprehension. *Journal of Reading Behaviour*, 15, 3–18.

60. Dockrell, J. E., & Messer, D. (2004). Lexical acquisition in the school years. In R. Berman (Ed.), Language development: *Psycholinguistic and typological perspectives.* New York: John Benjamins.

61. Parsons, S., Law, J., & Gascoigne, M. (2005). Teaching receptive vocabulary to children with specific language impairment: a curriculum-based approach. *Child Language Teaching and Therapy*, 21(1), 39-59.

62. Brigman, G. A., & Webb, L. D. (2003). Ready to Learn: Teaching Kindergarten Students School Success Skills. *Journal of Educational Research*, 96(5), 286-292.

This tool was developed as part of the Better Communication Research Programme 2012

About the Better Communication Research Programme:
The Better Communications Research Programme (BCRP) was a 3 year research programme. It was part of the government's response to the Bercow Review of provision for children and young people with speech, language and communication needs, published in July 2008.

The BCRP produced 17 reports and were published by the Department for Education in December 2012. During 2013-2015 the BCRP team and The Communication Trust are working together to share the findings and to use the research evidence to contribute to the development of policy and practice.

About The Communication Trust:
The Communication Trust is a coalition of nearly 50 voluntary and community organisations with expertise in speech, language and communication. We harness our collective expertise to support the children's workforce and commissioners to support all children and young people's communication skills, particularly those with speech, language and communication needs (SLCN).

We do this by raising awareness, providing information and workforce development opportunities, influencing policy, promoting best practice among the children's workforce and commissioning work from our members.

The Trust was founded in 2007 by children's charities Afasic and I CAN together with BT and the Council for Disabled Children.

The Communication Trust
Every child understood

www.thecommunicationtrust.org.uk

Published May 2013
