# Supplementary material for: Exploring the acceptability, feasibility, and appropriateness of a communication-friendly classroom tool for use in Irish schools: A qualitative inquiry
Source: PLoS One. 2023 Jun 22;18(6):e0287471. doi: 10.1371/journal.pone.0287471 (PMC10286983; doi:10.1371/journal.pone.0287471)
Supplement: S2 File — (PDF) [file pone.0287471.s002.pdf]

Participant background questionnaire

Questionnaire

1. **Which setting do you work in?** Primary/Post Primary
2. **What is your current role in the school?** SLT/Class teacher/ Subject Teacher/ Special Education Teacher/ Principal/ Deputy principal/ Special Class Teacher/ Resource Teacher
3. **How long have you been a teacher at your current school?**
4. **Is your school a designated DEIS school?** Yes/No
5. **How would you describe your school?** Urban/Rural
6. **How would you describe your school?** Mixed boys and girls/ Single sex boys/ Single sex girls
7. **How many students are in your school?**
8. **How many years teaching experience do you have?**
9. **Do you have any additional training and/or qualifications since graduating as a teacher? (short courses/post-graduate courses etc.)**
10. **Tell us about any additional information you think might be important for us to know.**

Semi-structured interview topic guide (practitioner)

School context

1. Can you tell me about your school/service? Prompts could include:
    - How many students/staff are in your school?
    - What is leadership like- principal, vice principal, year heads etc?
    - Are there opportunities for CPD?
    - Do you have opportunities to collaborate with an SLT?

Lesson planning

2. How and when do you usually plan your lessons? Prompts could include:
    - Do you use any planning tools in your teaching?
    - How do you plan for students with diverse needs?
    - How are you supported by the SEN team?

Using the checklist

3. Tell me about when you tried out the checklist? Prompts could include:

- When did you use it?
- Why did you choose that lesson to use it?
- How easy was it to get someone to use it with you?
- How did you feel about being observed/observing?

Opinions on the checklist

4. What did you think of the tool? Prompts could include:

- How long did it take you to use it?

- Was it easy to use? Why/Why not?
- Did you stick with the items or add/not use some items?
- Was the language accessible/easy to understand?
- Did it bring any new insights into how communication could be supported?
- What would you add/remove from the checklist?

Effectiveness of the tool

5. Did you and the other person using the tool identify any actions that you would follow up on for the classroom from the process?

- If yes, what were they?
- If no, why not?

Implementing the checklist in planning/teaching

6. Is the tool something you would use routinely in your practice?

   If yes, how, and when would you use it? What supports would you need to use it?

   If no, why not?

Implementing change in schools

In your school, if something new is suggested to happen, what are the most important factors to make that happen?

- What systems are in place in your schools to support your professional development?
- What personal factors do you think make it more likely that a teacher will implement change?
- Do you have any suggestions on what would be needed to embed a tool like this in school?

Conclusion: Is there anything else you want to tell me about that I haven't already asked, but that you feel is important?

Semi-structured interview topic guide (School Leaders)

General questions

1. How long have you been principal/ deputy principal here?
2. Tell me a little about your own teaching background- subjects, roles etc.
3. Do you enjoy your role?

School context

1. Tell me about the students at your school? Prompts could include:
   - How does the school/ individual teachers support students' learning?
   - How does the school/individual teachers meet diverse needs?
   - What models of provision are in place to support diverse student needs? E.g. in-class support/ co-teaching; small group withdrawal; individual withdrawal.

- How is provision for diverse learners/ those with additional needs/ SEN coordinated and led in the school?

2. Tell me about the ethos of the school- is there an inclusive ethos? How is this evident?
3. Do you think students with diverse/ additional needs are well served in the school? Are they making progress? How do you know?
4. In your opinion, what are the key benefits of including students with diverse needs in the school?
5. In your opinion, what are the key challenges to including students with diverse needs in your school?
6. In your opinion, how supportive are staff in general to implementing classroom level interventions?

School organisation

1. How are resources used in the school to respond to needs at whole-school level, class level and individual student level? Prompts could include:
    - How are SLCN identified in the school? How do you generate a school profile? (what types of assessments are undertaken).
    - Is there a core SEN team? How are additional resources deployed?
    - Is there a SENCO? Who leads and coordinates SEN provision?
    - How many SNAs are in the school and how are they deployed?
    - How does the SEN Team collaborate with class/ subject teachers to respond to SLCN at class level?
2. Do you think your school has enough resourcing to be able to meet the diverse range of needs in your school? Explain. Prompts could include:
    a. Is there support in the form of policy guidance?
    b. Is your additional resource allocation adequate?
    c. Does the school have sufficient access to external support agencies/ resources etc.?

Leading change in school

1. Have any new interventions been implemented in your school recently? Prompts could include:
    - What was the intervention?
    - Why did you implement it? What encouraged you to make those changes?
    - How has this experience been?
    - Who led the implementation strategy?

2. Are there systems in place to support staff in implementing new practices? Prompts could include:
    - Opportunities for staff to meet and discuss implications of the initiative- staff meetings for example.
    - Opportunities to problem solve together.
    - Opportunities for staff to share good practice, resources, and innovation both within and between schools.
    - Opportunities for staff to engage with CPD/ professional learning.
3. Based on your experiences, in your school, if something new is suggested to happen, what are the most important factors to make that happen?

Views of the CSCOT

1. Your school has participated in piloting the CSCOT tool. If this is found to be effective for Irish classrooms, and provides a useful guide for class/ subject teachers how might this work? Prompts could include:
    - Would you provide opportunities in staff meetings to discuss use of the tool?
    - Would you use SEN team have scheduled/ timetabled meetings?
    - Would you create opportunities for staff who have trialed the tool to cascade this across the school?
2. What would be needed to support the implementation of a tool like the CSCOT in your school?

Conclusion. Is there anything else you want to tell me about that I haven't already asked, but that you feel is important?
